# Improvement of derivatized amino acid detection sensitivity in micellar electrokinetic capillary chromatography by means of acid-induced pH-mediated stacking technique

**DOI:** 10.1007/s00216-014-8104-1

**Published:** 2014-08-22

**Authors:** Szymon Dziomba, Adrian Bekasiewicz, Adam Prahl, Tomasz Bączek, Piotr Kowalski

**Affiliations:** 1 Department of Pharmaceutical Chemistry, Medical University of Gdańsk, 107 Hallera Street, 80-416 Gdańsk, Poland; 2 Faculty of Electronics, Telecommunications and Informatics, Gdańsk University of Technology, 11/12 Gabriela Narutowicza Street, 80-233 Gdańsk, Poland; 3 Institute of Organic Synthesis, Department of Organic Chemistry, Faculty of Chemistry, University of Gdańsk, 63 Wita Stwosza Street, 80-952 Gdańsk, Poland

**Keywords:** Capillary electrophoresis, Human urine samples, On-line preconcentration techniques, Stacking, Sweeping

## Abstract

Derivatization is a frequently used sample preparation procedure applicable to the enhancement of analyte detection sensitivity. Amino acids mostly require derivatization prior to electrophoretic or chromatographic analysis, especially if spectrophotometric detection is used. This study presents an on-line preconcentration technique for derivatized amino acids. The sensitivity of the method was improved by the utilization of the proposed acid-induced pH-mediated stacking mechanism. The method is demonstrated by preconcentration of amino acids labeled with 2,4-dinitrofluorobenzene. Use of optimized conditions for a large sample volume injection (40 s, 13.8 kPa) followed by electrokinetic injection of 0.1 M HCl (20 s, 10 kV) gave a 20- to 30-fold enhancement of sensitivity. The significance of the sweeping mechanism and pseudo-isotachophoresis for the on-line sample focusing and the influence of parameters on the preconcentration process were discussed. The applicability of the elaborated method was demonstrated using human urine samples.

**Electronic supplementary material:**

The online version of this article (doi:10.1007/s00216-014-8104-1) contains supplementary material, which is available to authorized users.

## Introduction

Capillary electrophoresis (CE) is a separation technique that benefits from, e.g., substantial separation efficiency, fast analysis, and relatively low operation cost. Unfortunately, the small inner diameter of the capillary and consequently its short optical path significantly hinder spectrophotometric detection. This is a serious drawback, which results in higher detection limits than in high-performance liquid chromatography (HPLC), especially if injection of a small sample volume is required. On the other hand, a significant improvement of detection sensitivity in CE can be successfully obtained by means of sample injection techniques and careful selection of sample matrix and background electrolyte (BGE) composition [1–4].

A field-amplified stacking of charged molecules was one of the first reported methods of detection sensitivity enhancement for CE [5]. To date, many other preconcentration strategies have been developed. A number of interesting and comprehensive reviews on this topic have been published and revised [1–4]. A common approach to sample preconcentration is to generate a difference of conductivity between solution zones in the capillary. A high separation efficiency was obtained by Aebersold and Morrison by injection of peptides in a high pH matrix into capillary filled with acidic BGE [6]. This concept was further developed by Britz-McKibbin et al. into a so-called dynamic pH junction [7–9]. Another preconcentration approach involves the allocation of sample between acid and base zones [10]. The mechanism of this sandwich-type injection can be explained by the cross-titration of hydronium (H_3_O^+^) and hydroxyl (OH^−^) ions that facilitate stacking of analytes and amplifies the electric field by depletion of ions in the sample zone. Another preconcentration strategy, in which signal amplification is a result of H_3_O^+^ and OH^−^ titration (a so-called pH-mediated stacking), was efficiently implemented in a number of studies [11–19]. The technique is of great interest, especially owing to its compatibility with a mass spectrometry detector [12–19]. It generally benefits from a simple implementation and minimal preliminary sample preparation. The latter requires only sample dilution in an appropriate matrix, even for the analysis of biological fluids [13, 15, 19].

The advantages of pH-mediated stacking were utilized by Lunte et al. for preconcentration of organic cations in high ionic strength matrixes by electrokinetic injection (EKI) of sample instead of hydrodynamic injection (HD) [20, 21]. In pH-mediated acid stacking, which is a modification of the former method, field amplification is obtained by following the injection of the sample with EKI of strong acid (0.1 M HCl).The same scheme, yet related to the preconcentration of anions, was later reported by Xiong et al. [22]. They achieved a signal amplification effect by EKI of strong base (0.1 M NaOH) that titrated TRIS^+^ ions in the BGE zone. The pH-mediated acid/base-stacking technique was also verified by means of preconcentration of high ionic strength biological samples (e.g., microdialysate [23, 24] and microsomal incubations [25]).

Verification of the separation efficiency properties has been the subject of extensive research. Separation efficiency is greater for both acid and base stacking in the case of samples of high ionic strength [20, 26]. The best separation efficiency and peak heights were obtained for an acid to sample injection ratio of 1.6 [20, 27]; however, this relation depends on the mobility of analytes and BGE type [28]. The sensitivity may be additionally increased by eliminating a low-conductivity zone through application of a reversed pressure after sample stacking, which makes stacking effective even if non-titratable ions are present in the BGE [24, 29]. Arnett et al. [24] extended the stacking efficiency in the presence of BGE by utilization of increased ionic strength. Nonetheless, the pH value of the BGE exhibits an even greater effect on separation efficiency than the aforementioned technique [30]. Furthermore, the introduction of organic solvent into the sample (e.g., acetonitrile) can additionally increase the detection sensitivity [31].

Another parameter with a significant impact on the efficiency of CE is the injection mode of sample and acid/base, e.g., HD, EKI, and their combinations. An EKI of sample followed by EKI of strong acid/base was found to be the most effective for signal enhancement, owing to efficient mixing of analytes with titratable ions [28]. Moreover, EKI/EKI mode benefits from a longer part of the capillary being available for the separation. Other combinations, including HD of a sample followed by EKI of a strong base or HD/HD mode, were tested as well, yet they both suffer from a significant peak tailing [28]. Nevertheless, HD of a sample followed by HD of a base was successfully applied for the preconcentration of deoxyribonucleoside monophosphates [11]. The HD/HD mode allows one to avoid the dependency of amounts of injected analytes upon sample matrixes as well as unwanted electrolytic effects [11]. Moreover, HD of a sample benefits from simultaneous injection of acidic, basic, and neutral analytes [32]. Another advantage of HD injection mode lies in its higher precision in comparison to the EKI [33]. For that reason it may be considered as a simple technique, especially because it avoids the need to counterbalance electroosmotic flow during the EKI.

A contribution of isotachophoresis in the preconcentration mechanism of pH-mediated stacking was initially suggested by Schwer and Lottspeich [10]. A coupling of pH-mediated stacking and transient isotachophoresis (t-ITP) was then demonstrated by Baidoo et al. [12]. Recently, base- and acid-induced t-ITP methods for preconcentration of acidic and basic drugs under co-electroosmotic conditions were reported [34, 35]. The occurrence of a t-ITP process was shown by using experimental work as well as computer simulation data.

The aim of this work was to elaborate a novel and efficient on-line preconcentration technique of derivatized samples. A set of 20 biogenic amino acids labeled with 2,4-dinitrofluorobenzene (DNFB) were used as model drugs. The method proposed originates from acid-induced pH-mediated stacking technique; however here, a preconcentration effect was obtained by HD of a sample, previously diluted in titratable borate buffer after EKI of strong acid (0.1 M HCl). To the best of our knowledge the applicability of acid-induced pH-mediated stacking in preconcentration of organic anions has never been tested before. In this study, the analytes were efficiently stacked using the optimized conditions. Subsequently, micellar electrokinetic capillary chromatography (MECC) was utilized in order to separate them [36]. The synergism of the sweeping process and acid-induced pH-mediated stacking was discussed. The applicability of the method was demonstrated for the determination of amino acids in human urine samples.

## Experimental

### Apparatus and methods

All capillary electrophoresis experiments were carried out using two different systems. The preconcentration technique and amino acid derivatization procedure was carried out with a P/ACE 2100 device (Beckman Instruments, Fullerton, CA, USA) with UV detector (200 nm wavelength). The separation was conducted in an uncoated fused-silica capillary of 77 cm × 50 μm I.D. (Beckman). The method development and validation study were performed using a PA 800 Plus (Beckman) equipped with a diode array detector (360 nm wavelength). According to the vendor specification, the capillary exploited in the PA 800 Plus was 3 cm longer (80 cm × 50 μm I.D) than in P/ACE 2100. However, the effective capillary length in both systems was the same (70 cm). Cartridges and samples in both apparatus were thermostated at 25 °C. It should be noted that a good agreement of the results for both systems was obtained.

Measurements of ACN concentration were performed with a gas chromatograph (GC) equipped with a flame ionization detector (FID; Shimadzu, Kyoto, Japan) and a fused-silica capillary column coated with cross-linked 5 % phenyl/95 % dimethylpolysiloxane (30 m × 0.5 mm I.D. and 0.25 mm film thickness). The GC temperature program was as follows: initial temperature of 40 °C was held for 5 min, then it was increased to 260 °C at a rate of 20 °C/min and held for 5 min. The split ratio was 1:100. FID and injector temperatures were set to 280 °C. Moreover, helium with a flow of 1.10 mL/min was used as the carrier gas. The injection volume was 1 μL.

The pH value of the solutions was measured with a Beckman pH meter (Beckman), whereas incubation of the samples was performed in an Eppendorf Termomixer (Eppendorf, Hamburg, Germany). The number of theoretical plates was calculated using our in-house MATLAB-based program (P/ACE 2100) or 32 Karat software (32 Karat™ Version 8.0 Workstation, Beckman). The length and volume of the sample plug were calculated with CE Expert software (Beckman).

### Reagents and solutions

Sodium dodecyl sulfate (SDS; >98.5 %) and sodium tetraborate decahydate (borax; >99 %) were both purchased from Sigma Aldrich (St. Louis, MA, USA). 2-Amino-2-hydroxymethylpropane-1,3-diol (Tris; >99.8 %) and 0.1 M HCl solution were acquired from Bio-Rad Laboratories (Hercules, California, USA) and Beckman, respectively. Ringer’s solution, used as artificial urine, was purchased from Baxter (Deerfield, IL, USA). HPLC-grade methanol was obtained from VWR (Radnor, PA, USA). Finally, all amino acids were purchased from Merck (Darmstadt, Germany).

The stock solutions were prepared through dissolution of an appropriate amount of substances in deionized water (Basic 5, Hydrolab, Wislina, Poland). The following concentrations were utilized: 200 mM SDS, 200 mM Tris, and 100 mM borax. Moreover, a 2,4-dinitrofluorobenzene (Sigma Aldrich) solution was diluted to 25 mg/mL in methanol. BGE was prepared daily from stock solutions. The optimized BGE consisted of 140 mM SDS, 20 mM Tris, and 10 mM HCl (pH 8.20). The concentration of borate in each sample was 10 mM (cf. Sect. “Derivatization procedure”), unless stated otherwise. Furthermore, stock solutions of amino acids were prepared at a concentration of 500 μM. It should be stressed that aqueous solutions were utilized for experiments, whereas amino acids diluted in Ringer’s solution were used during the validation study. Aspartic acid and tyrosine (characterized by insufficient water solubility) were dissolved using the following mixtures: 1 M NaOH/water (1:5, v/v) and 1 M NaOH/water/methanol (1:3:2, v/v/v), respectively.

### General electrophoretic procedures

The procedure of new capillary conditioning involved the following sequence: 0.1 M NaOH (30 min), water (30 min), and BGE (10 min), whereas daily preparation was performed as follows: 0.1 M NaOH (10 min), water (10 min) BGE (10 min), and current conditioning (30 kV for 10 min). Moreover, the capillary was flushed with 0.1 M NaOH and water for 10 min at the end of each day. A pressure of 482.6 kPa (70 psi) was used during all cleaning procedures.

Electrophoresis was performed as follows: 2 min rinse of capillary with BGE, sample injection (13.8 kPa for 40 s) followed by subsequent EKI of 0.1 M HCl (10 kV for 20 s) and separation at a voltage of 30 kV. The capillary was rinsed with BGE (2 min) after each run. It should be emphasized that the water dipping procedure was applied between injections in order to avoid cross-contamination of buffer solutions and samples in vials.

### Derivatization procedure

The utilized procedure is an enhancement of previously reported methods [37, 38]. In the proposed approach, 10 μL of amino acid standards or properly pretreated (cf. Sect. “Urine samples preparation”) human urine sample, 10 μL of 100 mM sodium tetraborate solution, 78 μL of deionized water, and 2 μL of DNFB solution (25 mg/mL in methanol) were thoroughly mixed in a small Eppendorf tube and incubated at 60 °C for 40 min. The tenfold dilution of samples was necessary in order to eliminate the buffering effect during analysis of biological fluids. Subsequently, samples were vortexed, centrifuged, and stored in a fridge (4 °C) for at least 15 min to inhibit the reaction. Finally, CE analysis was performed.

### Urine sample preparation

Fresh urine samples were collected from six healthy male volunteers (24–33 years old), and stored in a refrigerator (−17 °C). The preparation procedure was as follows. Defrosted samples were centrifuged and then 200 μL of supernatant was mixed with 2 μL of homoarginine (500 μM; internal standard) and 600 μL of acetonitrile. Subsequently, the mixture was centrifuged and stored in a refrigerator (−17 °C) for at least 20 min in order to obtain separation of the phases. The upper phase (acetonitrile rich) was then removed with an automatic pipette, whereas the lower phase (acetonitrile depleted) was used for derivatization.

## Results and discussion

### Derivatization of analytes and preconcentration mechanism

Derivatization is a widespread pretreatment method for analysis of amino acids in CE, especially if UV or fluorescence detection is applied. Unfortunately, its application in CE is often hindered by poor sensitivity, particularly if UV detection is used. The aforementioned drawback may be partially alleviated by the utilization of in-capillary enrichment techniques. A detailed review on the topic was recently provided by Chiu [39]. The methods of on-line preconcentration were discussed elsewhere [40–43]. These techniques are based on stacking of a large sample volume, which leads to the sensitivity enhancement by about two orders of magnitude.

**Fig. 1:**
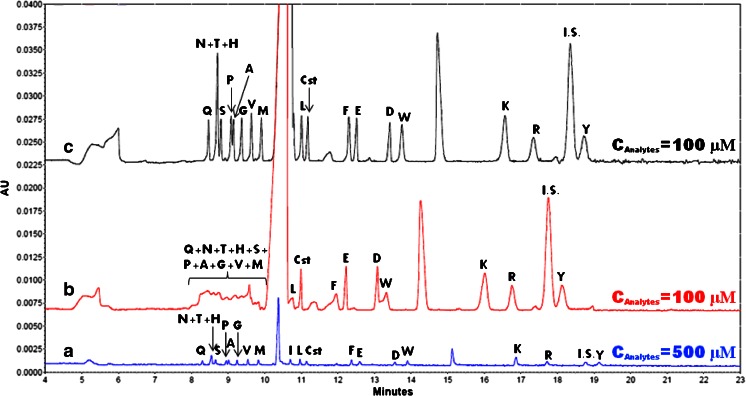
Comparison of electropherograms obtained by application of **a** hydrodynamic injection (5 s, 3.45 kPa) of sample (*c* = 500 μM); **b** injection of a large sample volume (40 s, 13.8 kPa; *c* = 100 μM); **c** injection of a large sample volume (40 s, 13.8 kPa; *c* = 100 μM) followed by electrokinetic injection of 0.1 M HCl (20 s, 10 kV). BGE, 140 mM SDS, 20 mM Tris, 10 mM HCl; uncoated fused silica capillary, 50 μm × 80 cm (70 cm effective length) thermostated at 25 °C; voltage, 30 kV; UV detection performed at 360 nm. Two DNFB peaks of high intensity can be observed at 10.5 and 14.5 min. *Q* glutamine, *N* asparagine, *T* threonine, *H* histidine, *S* serine, *P* proline, *A* alanine, *G* glycine, *V* valine, *M* methionine, *I* isoleucine, *L* leucine, *Cst* cysteine, *F* phenylalanine, *E* aspartic acid, *D* glutamic acid, *W* tryptophan, *K* lysine, *R* arginine, *Y* tyrosine, *I.S.* internal standard (homoarginine)

Borate is the most common buffer solution for performing the derivatization process under alkaline conditions. Unfortunately, a relatively high concentration of such a strong electrolyte in a sample disturbs stacking. Moreover, in contrast to a standard HD (5 s, 3.45 kPa; about 3.7 nL), the injection of a large sample volume into the capillary considerably deteriorates the separation efficiency. As a consequence, the peaks may not be completely separated (see Fig. 1a, b). For that reason, only signals of most retained compounds (e.g., basic amino acids) maintain their Gaussian peak shape due to the sweeping mechanism. High efficiency can be also obtained by increasing the hydrophobicity of cysteine (Cst), glutamic acid (Glu), and aspartic (Asp) acid, by means of their complexation with borate in a sample zone (see Fig. 1b) [44].

Despite the problems with high conductivity of derivatized samples, the electric field can be amplified in two ways: (i) by its dilution to the level at which stacking of analytes is efficient [42, 43], or (ii) by titration of the electrolyte in order to decrease conductivity. The former approach is inadequate for sample preconcentration, whereas the latter can be performed by sample injection into the capillary and subsequent EKI of strong acid (see Fig. 1c).

**Fig. 2:**
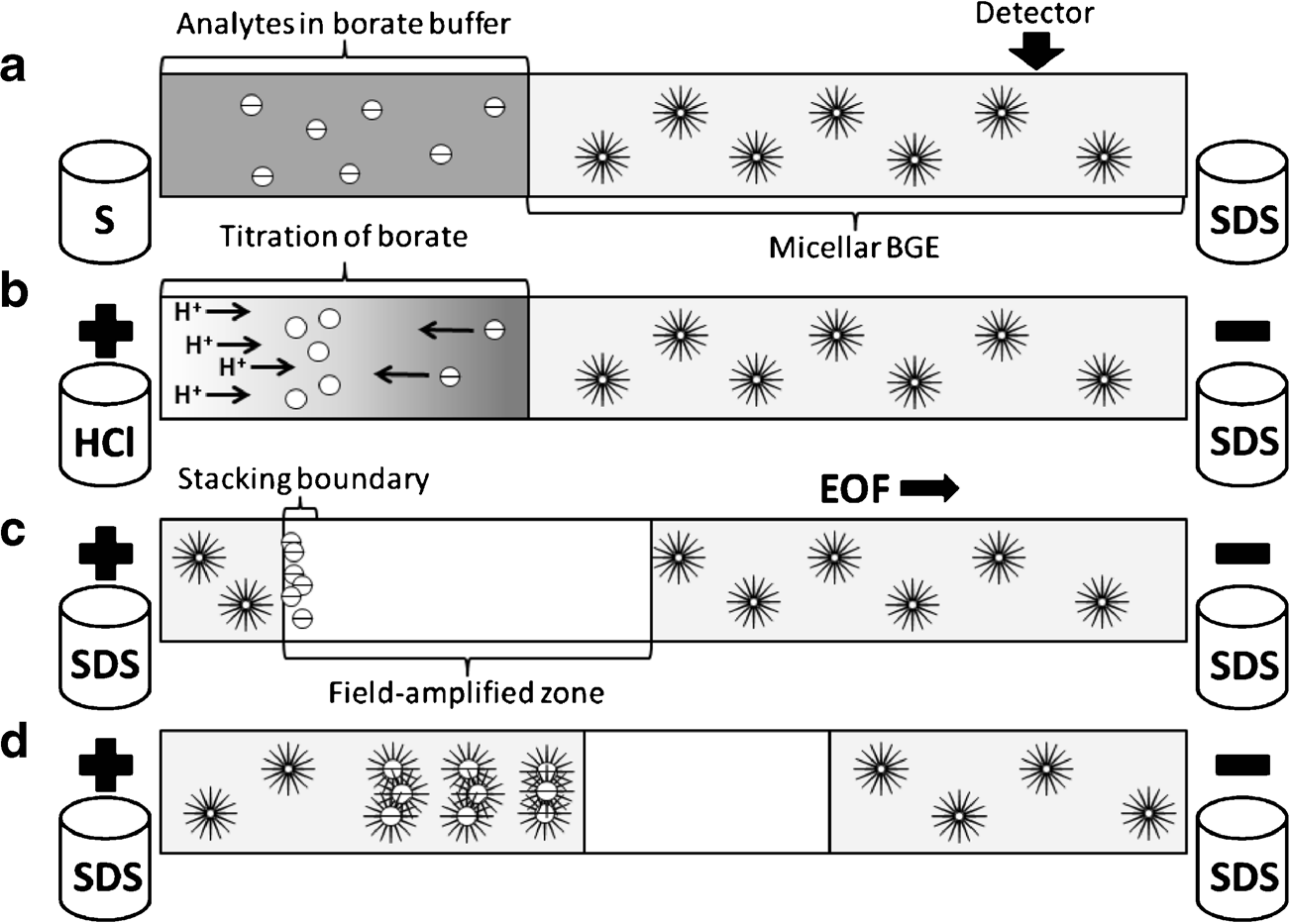
Preconcentration of analytes by means of acid-induced pH-mediated technique with hydrodynamic injection of sample. **a** Initially, the capillary was filled with micellar background electrolyte followed by hydrodynamic injection of a sample. The sample matrix contained borate buffer (*gray area*). **b** A vial with strong acid solution (0.1 M HCl) was situated at the inlet of the capillary and a high voltage was applied. Electromigration of hydronium ions into the capillary resulted in titration of borate, which was present in the sample zone (illustrated as lightening of the sample zone). **c** Conversion of borate ions into the poorly dissociated boric acid provided amplification of the electric field in the sample zone and stacking of analytes. **d** Finally, the micellar electrokinetic capillary chromatography was performed in order to separate the analytes. *Gray area* sodium tetraborate solution, *white area* boric acid solution

The preconcentration mechanism is illustrated in Fig. 2. Initially, a large sample volume was injected into the capillary that was preliminarily filled with micellar BGE (see Fig. 2a). Subsequently, a vial with strong acid (0.1 M HCl) was placed at the capillary inlet and a high voltage was applied (Fig. 2b). Electromigration of hydronium ions into the capillary resulted in titration of borate that was present in the sample zone and consequently formation of boric acid. The process promoted stacking by abrupt depletion of ionic compounds in the sample zone (Fig. 2c). Finally, micellar electrokinetic mode was utilized for separation of analytes (see Fig. 2d).

The discussed preconcentration process can be alternatively explained by the occurrence of the sweeping mechanism, because neutral specimens are more retained by the pseudostationary phase than the anionic ones [44]. However, an inverse relation was observed between the concentration of borate in the sample and improved separation efficiency. Therefore, electric field amplification in the sample zone through EKI of strong acid tends to be a more adequate explanation of this phenomenon. A detailed discussion on the concentration of separation buffer in the sample is given in Sect. “Parameters affecting the sensitivity enhancement”.

The protocols for sample derivatization that utilize DNFB reagent include addition of acetonitrile [37, 38], which considerably improves the solubility of DNFB in aqueous samples. On the basis of the conducted experiments, concentrations of ACN higher than 5 % (v/v) result in the occurrence of pseudo-isotachophoresis (p-ITP) [45]. Although the p-ITP was expected to enhance the detection sensitivity, an increased concentration of organic solvent in samples significantly influenced micelle stability at the boundary between sample and BGE, which resulted in weakening of the sweeping effect. It should be noted that the presence of ACN in the mixture was not essential for the DNFB-based derivatization process, but an extreme excess of DNFB in the sample resulted in residue formation. For that reason, we performed method optimization without addition of acetonitrile into derivatization mixture. Additionally, the preparation of urine samples was directed toward minimization of the acetonitrile concentration (cf. Sect. “Urine samples preparation”).

### Parameters affecting the sensitivity enhancement

Various parameters with a significant impact on the sensitivity of the proposed acid-induced pH-mediated stacking technique include the concentration of borate in the sample, length of injection plug, and type and concentration of acid used for stacking. It is noteworthy that these variables influence each other (e.g., the higher concentration of borate used in the sample, the more acid was required to be injected for titration).

The concentration of borate in the sample was considered an important parameter for sensitivity enhancement, mainly owing to the requirement of a high stability of pH value during derivatization of biological fluids, even in the presence of other buffering agents. The tests performed with a concentration of borate from 1 to 30 mM indicate that higher separation efficiency was obtained with lower borate concentrations owing to more efficient stacking of analytes. However, judging by the measurements, at least 5 mM borate was required to provide sufficient conditions for a stable and complete derivatization process. On the basis of the results obtained, 10 mM borate was selected for further experiments in order to enlarge the buffering capacity of the derivatization mixture.

Another parameter important in optimization was the time of acid injection. Analyte stacking was performed by EKI of 0.1 M hydrochloric acid at 10 kV. The acid injection was performed under the optimized conditions for 20 s, which made a complete titration of the injected sample plug possible (40 s, 13.8 kPa). The amount of acid needed to be injected depends on the concentration of borate as well as sample volume. An efficient stacking was obtained with 5 mM borate solution and a 10 s injection of HCl. One should emphasize that a very high separation efficiency can be obtained for 1 mM borate even without acid injection. The optimization of acid injection was found to be extremely important, because inefficient analyte stacking introduced by insufficient titration of the sample plug as well as an overlong HCl injection led to peak broadening.

The sample volume introduced into the capillary was considered as the most important parameter for sensitivity improvement. The highest signal amplification was obtained for 40-s sample injection at 13.8 kPa, which corresponds to 60.4 mm (118 nL) of the capillary length. Longer sample plugs caused a destacking effect and deterioration of separation efficiency.

Despite the unquestionable contribution of the sweeping mechanism to the signal enhancement effect, the concentration of SDS was also considered to be an important factor. The SDS level in the BGE primarily influenced the separation of analytes; thus, the manipulation of its concentration to achieve an increased sensitivity was strongly limited. Nonetheless, a considerable concentration of 140 mM SDS was found to provide the best separation of analytes. This relatively high amount of the surfactant provided a satisfactory sweeping effect; thus, the impact of SDS concentration on sweeping efficiency was not examined.

### Preliminary validation study

The performance of the elaborated method was validated under optimized separation conditions (cf. Sect. “Parameters affecting the sensitivity enhancement”) by calculation of respective coefficients, i.e., linearity, limit of detection (LOD), precision, and accuracy of the measurements. Calibration curves were constructed by analysis of six different concentrations of six independently prepared amino acid standards in Ringer’s solution. The results obtained indicated that the data fit the selected linear model well (see Table 1). Determination coefficients (*R* ^2^) greater than 0.9990 were obtained for the investigated concentration range. The calculated LOD values (based on signal to noise ratio S/N = 3) of the analytes varied from 1.5 μM (for lysine) to 3 μM (for other amino acids), whereas lower limits of quantification values (LLOQ)—determined for S/N = 10 for all analytes except lysine where S/N = 20—were set to 10 μM for all examined amino acids.

**Table 1:** Selected parameters obtained during the validation study

	Linearity range	Slope (*a*)	Intercept (*b*)	*R* ^2^	*N*/m (×1,000)
Gln (Q)	10–200 μM	474.35	0.0055	0.9994	274.1
Ser (S)		540.74	−1.0807	0.9989	172.1
Pro (P)		425.45	2.9922	0.9998	158.2
Ala (A)		627.02	−3.9044	0.9985	186.5
Gly (G)		432.20	0.4238	0.9995	174.7
Val (V)		421.67	1.0987	0.9998	193.8
Met (M)		476.38	3.0066	0.9999	198.2
Leu (L)		446.65	2.6677	0.9998	202.2
Phe (F)		461.91	4.9675	0.9993	117.4
Glu (D)		492.15	−1.914	0.9991	219.6
Asp (E)		507.18	−1.7482	0.9997	236.6
Trp (W)		558.78	1.756	0.9989	129.9
Lys (K)		255.71	0.3797	0.9999	137.1
Arg (R)		491.92	0.2299	0.9996	115.8
Tyr (Y)		487.68	2.3525	0.9996	97.6

*R* ^2^ determination coefficient, *N* number of theoretical plates (the calculation was performed for 3 runs using standard mixtures with a concentration of 100 μM)

Additionally, the homogeneity of the migration times and corrected peak area of amino acids were also examined. Intraday tests were conducted by analysis of six standard mixtures for four concentrations (10, 40, 80, and 160 μM), whereas interday results were obtained by performing three measurements a day for the same concentration level of analytes (10, 40, 80, and 160 μM) for three consecutive days. The obtained results indicated that the relative standard deviation (RSD) of the relative migration time was less than 2 %. The precision rates, expressed as RSD of calculated concentrations, varied from 0.54 % to 11.47 % and from 1.54 % to 11.07 % for intraday and interday experiments, respectively (see Electronic Supplementary Material (ESM), Table S1). The accuracy was assessed by means of recovery experiments conducted in the following manner. Known amounts of standard amino acids were added to Ringer’s solution. Then recovery values were calculated by comparison of obtained concentrations with added ones (see ESM, Table S2). One should stress that all calculated factors met the requirements of the European Medicines Agency for bioanalytical methods.

Finally, the sensitivity enhancement effect for analytes was calculated. Comparison of the peaks heights obtained for the standard HD method (5 s, 3.45 kPa) and optimized stacking technique revealed 20- to 32-fold improvement in signal intensities. Detailed results are given in ESM, Table S3. The sensitivity improvement of most of the assessed compounds ranged from 20 to 27, while the signal enhancement effect for aspartic and glutamic acids was found to be 32 and 31, respectively. This can be explained by the additional sweeping effect observed for these acidic compounds under non-stacking conditions (Sect. “Derivatization of analytes and preconcentration mechanism”). The effect of borate complexation of analytes and its impact on the sweeping efficiency needs further examination in the future.

### Application

**Fig. 3:**
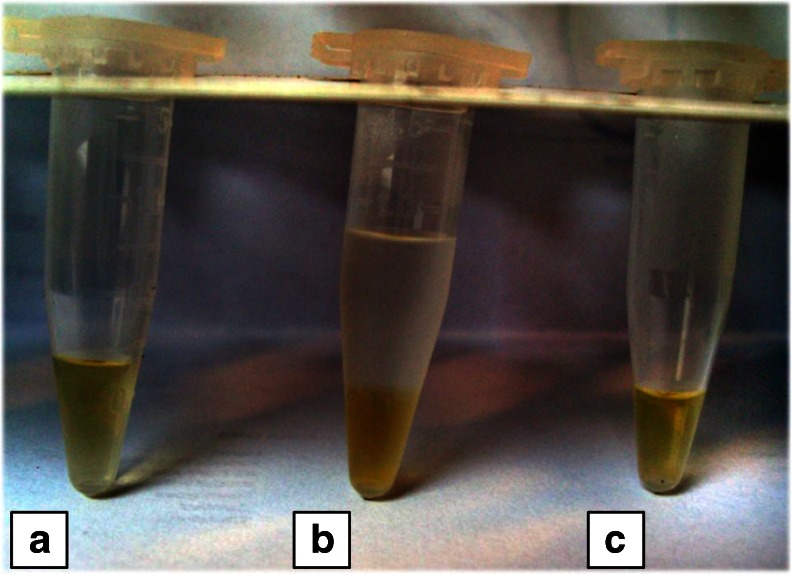
Procedure for urine sample preparation prior to CE analysis. **a** Urine sample mixed with I.S. was placed in an Eppendorf tube. **b** Deproteinization of urine using ACN was performed after cooling the sample to −17 °C, which resulted in a phase separation. **c** Urine sample with ACN-rich phase removed with a micropipette

**Fig. 4:**
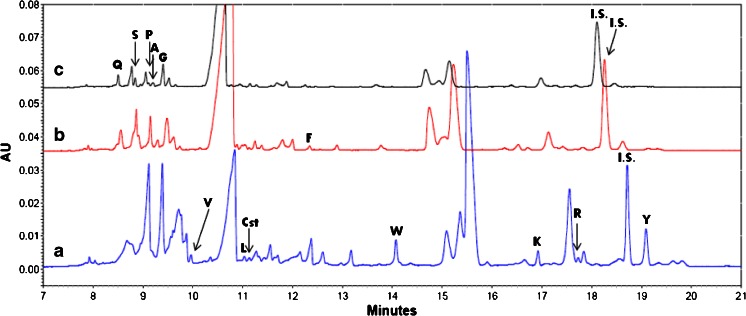
Electropherograms obtained through analysis of **a** urine sample; **b** urine sample diluted four times; **c** urine sample diluted eight times. Separation conditions remain the same as in Fig. 1c. The matched peaks have been used for quantitative analysis. Abbreviations are the same as in Fig. 1

A strong affinity between certain diseases and disrupted concentrations of amino acids in human biofluids has been observed over the years [15, 19, 46, 47]. The applicability of the elaborated method was shown using human urine samples, which were prepared according to the instructions provided in Sect. “Urine samples preparation”. In summary, the urine was deproteinized with acetonitrile by mixing them in a 1:3 (v/v) relation. Relatively high concentrations of acetonitrile in the prepared mixture destabilized micelles at the sample/BGE boundary during the analysis, which was considered to be an undesired effect (cf. Sect. “Derivatization of analytes and preconcentration mechanism”). Elimination of ACN may be easily achieved through evaporation; however, it significantly extends the sample preparation time and requires laboratory equipment. In this work, we utilized a simple and fast separation procedure (see Fig. 3). First, 200 μL of urine sample and 2 μL of I.S. were placed in the Eppendorf tube (see Fig. 3a) and mixed with 600 μL of ACN. Next, the mixture was stored at −17 °C in a typical laboratory refrigerator for a period of 20 min. This resulted in a phase separation into an aqueous (depleted of ACN) and organic one (Fig. 3b). The upper phase (rich in ACN) was removed using an automatic pipette (Fig. 3c). The concentration of ACN in the aqueous phase was about 30 % (v/v), which was sufficient for elimination of all undesired effects introduced by a large volume injection of the derivatized sample. The aqueous phase can be derivatized directly or after suitable dilution (cf. Sect. “Derivatization procedure”). The separation results of an exemplary urine sample are shown in Fig. 4 and the determined amino acids levels are given in Table 2. Application of the preconcentration method enabled the determination of low abundant amino acids, i.e., arginine, lysine, or valine (see Fig. 4a), whereas the quantification of the compounds of interest with considerably higher concentration required sample dilution (see Fig. 4c). Migration time shifts, visible between Fig. 4a–c, were compensated by the utilization of I.S. and application of relative migration times, which allowed for identification of signals. The quantification revealed the concentrations of some amino acids above the linearity range of the method (Table 2) as well as problems with the resolution according to the high concentration of analytes and matrix constituents (Fig. 4a). However, dilution of the sample enabled the determination of the most abundant compounds like glutamine, serine, alanine, glycine, and arginine. In the case of proline, the concentration was within the linearity of the method, although the signal was not separated from other compounds (Fig. 4a). Nevertheless, additional dilution of the sample enabled one to detect and quantify 13 amino acids. Methionine and aspartic acid were found to be below the limit of detection.

**Table 2:** Results of quantitative analysis of human urine samples obtained with elaborated stacking technique (*n* = 6)

Amino acid	Determined concentration range (μM)
Gln	272–619
Ser	156–404
Pro	70–148
Ala	156–293
Gly	430–990
Val	12–53
Met	Not detected
Leu	23–59
Phe	57–99
Glu	<LOQ–46
Trp	39–161
Asp	Not detected
Lys	34–128
Arg	37–246
Tyr	101–197

## Conclusion

In this study an acid-induced pH-mediated stacking technique was used to increase the detection sensitivity of analytes in a sample with relatively high concentration of borate. The introduced methodology provides a field amplification in the sample zone by means of on-line electrokinetic injection of strong acid. The proposed technique outperforms a conventional approach based on the off-line injection of acid into the sample prior to the analysis, because it significantly limits the amount of ions in the amplification zone. The problem with separation and preconcentration of samples in the presence of relatively high concentrations of ACN in MECC was addressed by our simple and reproducible methodology based on mechanical ACN elimination from aqueous samples. The method was successfully demonstrated, which showed its vital usefulness for the improvement of detection sensitivity of derivatized amino acids in micellar electrokinetic capillary chromatography. Moreover, the proposed methodology should be applicable to the on-line preconcentration of other specimens containing weak acid as a buffering agent. To the best of our knowledge, this is the first successful attempt to realize the preconcentration of organic anions in micellar electrokinetic capillary chromatography by means of acid-induced pH-mediated stacking.

## Electronic supplementary material

Below is the link to the electronic supplementary material.

ESM 1 (PDF 28 kb)
