# Supplementary material for: Improvement of derivatized amino acid detection sensitivity in micellar electrokinetic capillary chromatography by means of acid-induced pH-mediated stacking technique
Source: Anal Bioanal Chem. 2014 Aug 22;406(26):6713–21. doi: 10.1007/s00216-014-8104-1 (PMC4182592; doi:10.1007/s00216-014-8104-1)
Supplement: Supplementary file 1 — (PDF 28 kb) [file 216_2014_8104_MOESM1_ESM.pdf]

Analytical and Bioanalytical Chemistry

Electronic Supplementary Material

Improvement of derivatized amino acids detection sensitivity in micellar electrokinetic capillary chromatography by means of acid-induced pH-mediated stacking technique

Szymon Dziomba, Adrian Bekasiewicz, Adam Prahl, Tomasz Bączek, Piotr Kowalski

Table S1 Comparison of intra- and inter-day precision values obtained for various concentrations (C) of analytes

	Precision							
	Intra-day (% RSD, n=6)				Inter-day (% RSD, n=9)			
C [μM]	160	80	40	10	160	80	40	10
**Gln (Q)**	2.05	3.45	7.42	11.47	3.92	5.89	5.57	9.91
**Ser (S)**	2.29	2.05	5.43	2.89	1.54	6.47	5.24	5.38
**Pro (P)**	2.26	1.03	4.81	6.00	3.12	5.22	4.59	11.07
**Ala (A)**	3.99	2.87	3.15	8.59	4.11	7.04	7.33	8.85
**Gly (G)**	2.20	3.12	3.57	4.03	2.13	3.25	4.21	9.65
**Val (V)**	3.14	5.10	5.47	6.83	2.59	4.59	6.22	6.15
**Met (M)**	4.36	1.93	7.50	9.90	3.91	6.99	8.20	9.90
**Leu (L)**	2.24	2.29	1.88	4.10	1.59	2.41	7.47	8.50
**Phe (F)**	0.54	1.19	2.88	5.67	1.37	3.50	3.90	5.15
**Glu (D)**	5.21	2.70	1.58	5.90	3.67	6.46	3.78	9.31
**Asp (E)**	2.42	3.03	4.51	7.26	3.44	6.56	3.72	4.96
**Trp (W)**	4.86	6.48	4.51	8.42	4.48	7.45	5.26	9.87
**Lys (K)**	0.84	2.49	0.85	5.13	2.79	5.09	2.15	4.15
**Arg (R)**	0.67	3.31	2.67	4.70	2.96	3.86	3.34	4.54
**Tyr (Y)**	1.70	2.07	2.13	5.23	2.03	4.89	3.04	5.03

Table S2 Comparison of intra- and inter-day values of accuracy obtained for various concentrations (C) of analytes

	Accuracy							
	Intra-day (n=6)				Inter-day (n=9)			
C [μM]	160	80	40	10	160	80	40	10
**Gln (Q)**	93.0	96.2	99.1	95.4	94.0	92.1	100.8	95.9
**Ser (S)**	93.0	98.4	96.5	108.1	92.7	97.5	96.6	112.1
**Pro (P)**	93.3	100.3	106.3	111.1	94.7	105.4	109.6	114.1
**Ala (A)**	96.3	101.3	98.5	88.8	95.0	94.5	92.2	86.7
**Gly (G)**	97.4	107.0	110.8	94.0	96.5	110.3	112.6	96.8
**Val (V)**	95.0	100.6	108.4	101.2	95.5	105.5	109.0	102.2
**Met (M)**	93.8	96.5	97.4	116.0	92.2	102.9	96.7	116.0
**Leu (L)**	90.4	95.8	104.7	102.5	90.4	96.8	103.5	102.5
**Phe (F)**	89.6	96.9	105.4	114.9	88.8	100.2	108.4	116.9
**Glu (D)**	96.9	100.2	91.8	84.9	95.6	93.7	89.0	84.2
**Asp (E)**	92.5	96.7	88.8	90.4	93.8	90.4	86.8	90.1
**Trp (W)**	96.8	94.7	108.2	107.7	87.3	95.9	110.3	117.2
**Lys (K)**	98.1	100.1	93.8	92.3	95.0	95.2	92,00	92.0
**Arg (R)**	97.5	100.0	96.2	92.3	94.2	97.6	95.5	91.6
**Tyr (Y)**	93.3	97.8	103.5	114.1	92.3	102.8	105.7	115.6

Table S3 The signal enhancement effect calculated on the basis of peak heights obtained for two injection modes: standard hydrodynamic injection (5 s, 3.45 kPa) for analytes at the concentration of 500 μM and elaborated preconcentration technique for analytes at the concentration of 100 μM (n = 3). Height values were expressed as counts

	Preconcentration	Standard injection	Signal enhancement*
**Gln**	4426	868	25
**Ser**	4460	912	24
**Pro**	4563	846	27
**Ala**	4510	944	24
**Gly**	4687	974	24
**Val**	5197	1098	24
**Met**	4701	998	24
**Leu**	4942	1070	23
**Phe**	4800	1088	22
**Glu**	4694	754	31
**Asp**	4233	670	32
**Trp**	3959	922	21
**Lys**	4957	1214	20
**Arg**	2589	630	21
**Tyr**	2801	582	24

* Signal enhancement effect was expressed as peak heights ratio for two tested methods multiplied with 5 (dilution factor)
